# The role of immune checkpoint inhibition in the treatment of ovarian cancer

**DOI:** 10.1186/s40661-016-0033-6

**Published:** 2016-11-24

**Authors:** Stéphanie L. Gaillard, Angeles A. Secord, Bradley Monk

**Affiliations:** 1 Department of Medicine, Division of Medical Oncology, Duke Cancer Institute, 200 Trent Drive, Durham, NC 27710 USA; 2 Department of Obstetrics and Gynecology, Division of Gynecologic Oncology, Duke Cancer Institute, 200 Trent Drive, Durham, NC 27710 USA; 3 Department of Obstetrics and Gynecology, Division of Gynecologic Oncology, University of Arizona College of Medicine, 2222 E. Highland Ave., Suite 400, Phoenix, AZ 85016 USA

**Keywords:** Ovarian cancer, Fallopian tube cancer, Primary peritoneal cancer, Immunotherapy, Immune checkpoint inhibitors, PD-1, PD-L1, CTLA-4

## Abstract

The introduction of immune checkpoint inhibitors has revolutionized treatment of multiple cancers and has bolstered interest in this treatment approach. So far, emerging clinical data show limited clinical efficacy of these agents in ovarian cancer with objective response rates of 10–15% with some durable responses. In this review, we present emerging clinical data of completed trials of immune checkpoint inhibitors and review ongoing studies. In addition we examine the current knowledge of the tumor microenvironment of ovarian cancers with a focus on the significance of PD-L1 expression and tumor-infiltrating lymphocytes on predicting response to immune checkpoint blockade. We evaluate approaches to improve treatment outcomes through the use of predictive biomarkers and patient selection. Finally, we review management considerations including immune related adverse events and response criteria.

## Background

### Role of immune checkpoints and development of immune checkpoint inhibitors

Ovarian cancer is the most lethal of the gynecologic malignancies. Over 22,000 new cases of ovarian cancer are diagnosed each year in the United States resulting in greater than 14,000 deaths per year [1]. The five year survival rate is less than 25% for women diagnosed with advanced stage disease (stage III or IV) despite aggressive treatment with surgery and adjuvant chemotherapy. Although >80% of patients will have a response to initial therapy, epithelial ovarian cancer ultimately recurs in the majority of patients. Recurrence is associated with a poor prognosis because of the eventual development of chemotherapy-resistant disease. Thus there is a great need, and opportunity, to improve ovarian cancer outcomes by understanding the immune milieu of ovarian cancers and harnessing the power of immunotherapy. This review will focus on the current understanding of the immune microenvironment of ovarian cancers and the potential role for immunotherapy in the treatment of this disease.

Immunotherapy refers to treatment designed to enhance an individual’s own immune function to eradicate malignant cells. While there have been various approaches, from cancer vaccines to adoptive immune cell therapies, immune checkpoint inhibitors have caused a paradigm shift in cancer treatment. These therapies are now FDA-approved for a variety of cancers including melanoma, non-small cell lung cancer (NSCLC), renal cell carcinomas (RCC), bladder cancer, and classical Hodgkin lymphoma. The enthusiasm for this approach stems from evidence of complete and long-lasting tumor regression in malignancies that are often refractory to chemotherapy.

T-cell mediated cancer cell death requires the production of effector T-cells (T_eff_) through the coordinated initiation of a multi-step process involving antigen presentation, priming and activation, T-cell trafficking and infiltration into the tumor, recognition of cancer cells, and cancer cell elimination [2]. This T-cell mediated immune response is regulated by a number of stimulatory and inhibitory signals. Inhibitory signals serve to prevent pathologic over-activation of the immune system, as an uncontrolled inflammatory response could result in the development of autoimmune or inflammatory disorders. However, inhibition of the T_eff_ response against cancer cells contributes to immune evasion. These inhibitory signals may come from extrinsic sources, such as regulatory T-cells (T_regs_) and inhibitory cytokines, or intrinsic sources, such as immune checkpoint proteins expressed on the surface of T_eff_. It is the balance of these signals that determines the success or failure of the immune system to eliminate cancer cells.

T_regs_ play a critical role in the extrinsic suppression of anti-tumor immunity. When T_regs_ are the dominant T-cell population in the tumor microenvironment, they inhibit tumor-antigen specific immunity and promote tumor growth. Depletion of these T_regs_ can restore anti-tumor immune activity. Similarly, other suppressive immune cells [e.g. myeloid derived suppressor cells (MDSCs), M2 macrophages] influence the balance of regulatory signals.

**Fig. 1:**
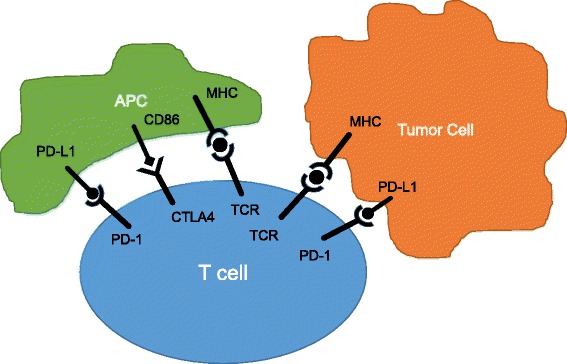
Costimulatory and coinhibitory pathways regulate the T-cell response to antigen. APC: antigen-presenting cell, CTLA-4: cytotoxic T lymphocyte-associated protein 4, MHC: major histocompatibility complex, PD-1: programmed cell death protein 1; PD-L1: PD-1 ligand, TCR: T-cell receptor

Immune checkpoint receptors, such as cytotoxic T lymphocyte-associated protein 4 (CTLA-4) and programmed cell death protein 1 (PD-1), have emerged as critical intrinsic modulatory mechanisms impairing natural anti-neoplastic immunity (Fig. 1). These receptors are negative regulators which attenuate normal T-cell activation to prevent pathologic over-activation. Interfering with immune checkpoint signaling has been shown to enhance anti-tumor immune responses through the recovery of T-cell function. The CTLA-4 and PD-1 immune checkpoint proteins function at different points in the process, which may explain their differential activities and toxicities. The CTLA-4 immune checkpoint regulates T-cell priming and activation, activities that occur in the early phases of the immune response. Inhibition of CTLA-4 during the T-cell priming/ activation step leads to dysregulated expansion of auto-reactive T cells, including tumor-specific T-cells. Anti-CTLA-4 inhibitors have been associated with significant immune-related toxicities which are likely a result of the indiscriminate and unselected activation of auto-reactive T-cells.

PD-1 is a cell surface receptor that is upregulated during normal T-cell activation and modulates the activity of antigen-experienced effector T-cells. Interaction of PD-1 with either of its two known ligands, PD-L1 and PD-L2, results in inhibition of T-cell signaling and cytokine production as well as decreased effector T-cell numbers due to limited T-cell proliferation and increased susceptibility to apoptosis. Of the two ligands, PD-L1 appears to be the more relevant in the tumor microenvironment and is expressed on a wide range of tumor cells. Tumor-infiltrating lymphocytes can induce PD-L1 expression on tumor cells leading to reduced anti-tumor immunity [3]. PD-L1 expression may also be regulated through gene amplification or via oncogenic signaling pathways [4–6]. Antibodies directed at either PD-1 or PD-L1 result in abrogation of the negative signal, thus restoring T-cell function.

An important distinction between CTLA-4 and PD-1/L1 inhibitors is their location of action [7]. Because CTLA-4 regulates T-cell priming and activation, anti-CTLA-4 antibodies lead to activation of T-cells in lymphoid peripheral tissues. Anti-PD-1/L1 effects appear to be limited to the tumor microenvironment without evidence of recirculation. Thus a number of pharmacodynamic markers for anti-CTLA-4 activity have been identified in the peripheral blood, whereas no biomarkers of PD-1 activity have been isolated from peripheral blood thus far.

### Evidence for using checkpoint inhibitors in ovarian cancer

Two central tenets have emerged to predict effective treatment with immune checkpoint inhibitors: 1) accessibility of the tumor by effector immune cells and 2) dominance of the immune checkpoint pathways as the mechanism suppressing anti-tumor immunity. The first is frequently defined by the presence of tumor-infiltrating lymphocytes or the ratio of effector immune cells [i.e. T_eff_, dendritic cells (DCs), M1 macrophages] to immune suppressive immune cells [i.e. T_regs_, myeloid derived suppressor cells (MDSCs), M2 macrophages]. The second principle is less well defined as no accurate biomarker has been identified, although multiple approaches are being evaluated. Expression of PD-L1 on tumor cells has been suggested as a predictive biomarker to identify cancers that may be more responsive to PD-1/PD-L1 inhibitors [8]. Based on work initially performed in melanomas, tumors have been classified into 4 groups based on the presence of tumor-infiltrating lymphocytes (TILs) and PD-L1 expression (Table 1) [3, 9]. Type I tumors exhibit a pattern of adaptive immune resistance and may be most likely to respond to immune checkpoint inhibitors. Conversely, Type II tumors show no discernable immune reaction and single agent checkpoint blockade is unlikely to be successful. Alternative approaches that include methods to recruit effector immune populations to the tumor (e.g. vaccines), possibly in combination with immune checkpoint inhibitors, are predicted to be necessary. Type III tumors exhibit intrinsic expression of PD-L1, possibly through oncogenic stimulation, with no immune reactivity. This highlights that tumor expression of PD-L1 alone cannot be used as an indicator of potential benefit of PD-1/L1 inhibition as without effector immune cells in the tumor, single agent immune checkpoint inhibition is unlikely to be beneficial. Similar to type II tumors, approaches to stimulate immune trafficking to the tumor will be necessary. Finally Type IV tumors display a pattern of tolerance to immune infiltration that is not dependent on PD-L1 expression. Thus, other suppressive signals are likely present and inhibition of other checkpoint receptors may be beneficial. Although this stratification system is based on studies in melanoma and presents several caveats (ref. [9]), it provides a framework for understanding the tumor microenvironment and rationale for the benefit of immune checkpoint inhibitors in ovarian cancer.

**Table 1:** Classification of tumors based on presence of tumor infiltrating lymphocytes (TIL) and PD-L1 expression (based on Teng et al. [9])

Type I: Adaptive immune resistance TIL+ PD-L1+	Type II: Immunological ignorance TIL- PD-L1-
Type III: Intrinsic induction TIL- PD-L1+	Type IV: Tolerance TIL+ PD-L1-

### Prognostic significance of ovarian cancer tumor microenvironment

Evidence of the importance of the local tumor immune microenvironment in ovarian cancer emerged in 2003 when Zhang et al. showed that infiltration of treatment naïve tumors with T-cells was associated with a significantly improved median progression free (22.4 vs 5.8 months, *p* < 0.001) and overall survival (50.3 vs 18.0 months, *p* < 0.001) compared to tumors with no T-cells present [10]. However, we have since learned that not only is presence of T cells important, but that the type of T-cell present influences outcomes. The proportion of T_regs_ in the tumor negatively impacts clinical outcomes and was a predictor of increased risk of death in a multi-variate analysis [11, 12]. Multiple studies have since confirmed that the ratio of immune suppressive to effector immune infiltrates within ovarian tumors is associated with clinical outcome [13–17]. Immune responses to ovarian cancer appear to vary by histologic subtype with high-grade serous cancers most likely associated with a prognostically favorable tumor-infiltrating lymphocyte response [18, 19]. Classification of different histologic subtypes of ovarian cancers based on TIL and PD-L1 revealed that type I patterns were more common in high-grade serous cancers while type IV patterns predominated in other histologic subtypes (Table 2) [18].

**Table 2:** Classification of ovarian cancers by type of immune microenvironment (based on Webb et al. [18])

		% Total for histologic subtype			
Histologic subtype	*N*	*Type I*	*Type II*	*Type III*	*Type IV*
High-grade serous	112	57.4	5.1	0	37.4
Low-grade serous	11	0	9.1	0	90.9
Mucinous	30	26.7	16.7	0	56.7
Endometrioid	125	22.4	14.4	1.6	61.6
Clear cell	129	16.2	30.2	0	53.5

### Expression of PD-L1 on ovarian cancer cells

Hamanishi and colleagues first reported that high expression of PD-L1 on ovarian cancer cells was associated with poorer outcomes [20]. The 5-year survival rate for patients with high- versus low-expressing PD-L1 tumors was 52.6 ± 7.7% versus 80.2 ± 8.9%, *p* = 0.016, respectively. PD-L2 expression was also associated with poorer outcomes but was not statistically significant. High expression of PD-L1 on ovarian cancer cells was associated with reduced infiltration of cytotoxic T lymphocytes into tumors suggesting that PD-L1 expression promotes an immunosuppressive microenvironment by inhibiting T-cell infiltration [20]. Both PD-L1 expression and TIL were independent prognostic factors, though PD-L1 expression was inversely correlated with survival. In pre-clinical models, PD-L1 expression can be induced by interferon-gamma (often produced by TILs) and administration of chemotherapy, suggesting a balance that maintains an immune suppressive environment [21, 22]. PD-1/L1 blockade causes regression of ovarian tumors in a syngeneic ovarian cancer mouse model further validating the importance of this regulatory pathway [23].

PD-L1 expression is not limited to tumor cells and has been reported on immune cells including antigen-presenting cells, T-cells, and B-cells. A recent study showed that PD-L1 expression is predominantly expressed by macrophages in ovarian cancer rather than on the ovarian cancer cells themselves; in this context, macrophage associated PD-L1 expression was a marker of favorable prognosis [18]. The differences between this study and the one above may be due to the differences in the antibodies used, but also reflect the developing understanding of PD-L1 expression and its prognostic role in ovarian cancer. PD-L1 expression may be a marker of a tumor poised to respond to immune stimulatory effects of chemotherapy or perhaps because PD-L1 may suppress the activity of immune-suppressive immune cells (i.e. T_regs_), PD-L1 expression on immune cells could tip the balance towards a more favorable immune microenvironment [24]. Thus evaluating PD-L1 expression on tumor cells in isolation is not sufficient to predict immune response and efficacy of immune checkpoint blockade in ovarian cancer.

### Trials of immune checkpoint inhibitors in ovarian cancer

**Fig. 2:**
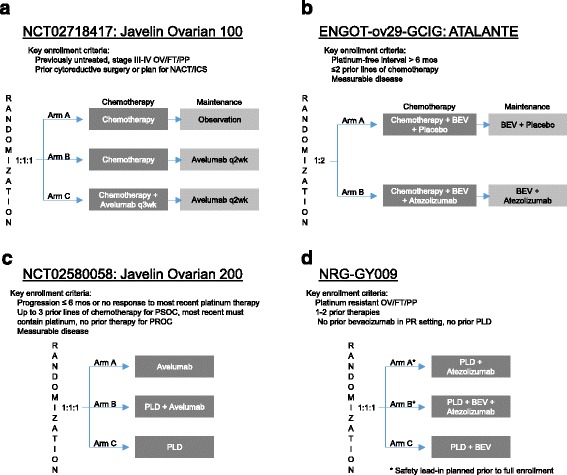
Ongoing or planned phase 3 trials in ovarian cancer with immune checkpoint inhibitors. **a** NCT02718417: Javelin Ovarian 100. **b** ENGOT-ov29-GCIG: ATALANTE. **c** NCT02580058: Javelin Ovarian 200. **d** NRG-GY009

Several antibodies directed against PD-1, PD-L1, and CTLA-4 have been developed and are being tested clinically in patients with ovarian cancer. Table 3 reflects the latest data from studies that have reported outcomes. Table 4 shows ongoing ovarian cancer trials with immune checkpoint inhibitors as monotherapy or combined with other agents. The schema for ongoing or planned phase 3 studies are shown in Fig. 2.

**Table 3:** Studies of immune checkpoint inhibitors in ovarian cancer with reported results

Immunotherapy agent(s)	Trial number	Disease status	Phase	N	Results (N; duration)	G3/4 adverse events	Reference
Ipilimumab		recurrent EOC, previously treated with GVAX vaccine	I	9	PR (1; 35+ mos.) SD (3; 1 for 6+ mos.)	diarrhea	Hodi et al. [50]
BMS-936559 (anti-PD-L1)	NCT00729664	recurrent EOC	I	17	6% PR (1; 1.3+ mos.) 18% SD (3; 6+ mos.)	infusion-related reaction, adrenal insufficiency	Brahmer et al. [80]
Nivolumab		platinum resistant EOC	II	20	10% CR (2; 11+ mos.) 5% PR (1; 11+ mos.) 30% SD (6; 1 for 11+ mos.)	lymphocytopenia, hypoalbuminemia, elevated ALT, rash, fever, anemia	Hamanishi et al. [25]
Pembrolizumab	NCT02054806	recurrent EOC, PD-L1 positive	Ib	26	4% CR (1; 6+ mos.) 8% PR (2; 6+ mos.) 23% SD (8; 2 for 6+ mos.)	transaminitis	Varga et al. [26]
Ipilimumab	NCT01611558	recurrent EOC	II	40	10% BRR (4; NA)	NA	clinicaltrials.gov [27]
Avelumab	NCT01772004	recurrent EOC	Ib	124	10% PR (12; 4 for 6+ mos.) 44% SD (55; NA)	rash, edema, elevated amylase/lipase, arthritis, colitis, hyperglycemia/DM	Disis et al. [28]
Durvalumab + Olaparib	NCT02484404^a^	recurrent EOC	I/II	10	PR (1; 11+ mos.) SD (7; 4+ mos.)	Lymphopenia, anemia	Lee et al. [29]
Durvalumab + Cediranib				4	PR (1; 7 mos.) SD (2; 1 for 6 mos.)	Lymphopenia, anemia, nausea, diarrhea, hypertension, PE, pulmonary hypertension, fatigue, headache	

*Abbreviations: N* number of ovarian cancer patients treated, *EOC* epithelial ovarian cancer, *CR* complete response, *PR* partial response, *SD* stable disease, *ALT* alanine aminotransferase, *BRR* best response rate (CR/PR status not provided), *mos*. months, *NA* not available, *DM* diabetes mellitus; PE, pulmonary embolism

^a^As of data cut-off date: May 10, 2016

**Table 4:** Ongoing studies of immune checkpoint inhibitors in ovarian cancer

Phase	Trial number	Trial	Disease status	Immunotherapy agent(s)	Concurrent therapy
3	NCT02580058	A Study Of Avelumab Alone Or In Combination With Pegylated Liposomal Doxorubicin Versus Pegylated Liposomal Doxorubicin Alone In Patients With Platinum Resistant/Refractory Ovarian Cancer (JAVELIN Ovarian 200)	recurrent platinum resistant	Avelumab	Liposomal Doxorubicin
3	NCT02718417	Avelumab in Previously Untreated Patients With Epithelial Ovarian Cancer (JAVELIN OVARIAN 100)	primary	Avelumab	Carboplatin Paclitaxel
3	ENGOT-ov29-GCIG	A randomized, double-blinded, phase III study of atezolizumab versus placebo in patients with late relapse of epithelial ovarian, fallopian tube, or peritoneal cancer treated by platinum-based chemotherapy and bevacizumab	recurrent platinum sensitive	Atezolizumab	Carboplatin-based chemotherapy Bevacizumab
2	NCT02440425	Dose Dense Paclitaxel With Pembrolizumab (MK-3475) in Platinum Resistant Ovarian Cancer	recurrent platinum resistant	Pembrolizumab	Dose Dense Paclitaxel
2	NCT02498600	Nivolumab With or Without Ipilimumab in Treating Patients With Persistent or Recurrent Epithelial Ovarian, Primary Peritoneal, or Fallopian Tube Cancer	recurrent platinum sensitive/resistant	Nivolumab +/- Ipilimumab	
2	NCT02520154	Pembrolizumab in Combination With Chemotherapy in Frontline Ovarian Cancer	primary	Pembrolizumab	Carboplatin Paclitaxel
2	NCT02659384	Anti-programmed Cell Death-1 Ligand 1 (aPDL-1) Antibody Atezolizumab, Bevacizumab and Acetylsalicylic Acid in Recurrent Platinum Resistant Ovarian Cancer	recurrent platinum resistant	Atezolizumab	Bevacizumab Acetylsalicylic Acid
2	NCT02674061	Efficacy and Safety Study of Pembrolizumab (MK-3475) in Women With Advanced Recurrent Ovarian Cancer (MK-3475-100/KEYNOTE-100)	recurrent platinum sensitive/resistant	Pembrolizumab	
2	NCT02764333	TPIV200/huFR-1 (A Multi-Epitope Anti-Folate Receptor Vaccine) Plus Anti-PD-L1 MEDI4736 (Durvalumab) in Patients With Platinum Resistant Ovarian Cancer	recurrent platinum resistant	Durvalumab	TPIV200/huFR-1 (anti-folate receptor vaccine)
2	NCT02766582	Phase II: Pembrolizumab/Carboplatin/Taxol in Epithelial Ovary Cancer	suboptimally cytoreduced primary	Pembrolizumab	Carboplatin Paclitaxel
1/2	NCT02431559	A Phase 1/2 Study of Motolimod (VTX-2337) and MEDI4736 in Subjects With Recurrent, Platinum-Resistant Ovarian Cancer for Whom Pegylated Liposomal Doxorubicin (PLD) is Indicated	recurrent platinum resistant	Durvalumab	Motolimod Pegylated Liposomal Doxorubicin
1/2	NCT02484404	Phase 1 and 2 Study of MEDI4736 in Combination With Olaparib or Cediranib for Advanced Solid Tumors and Recurrent Ovarian Cancer	recurrent platinum sensitive/resistant	Durvalumab	Olaparib or Cediranib
1/2	NCT02485990	Study of Tremelimumab Alone or Combined With Olaparib for Patients With Persistent EOC (Epithelial Ovarian, Fallopian Tube or Primary Peritoneal Carcinoma)	recurrent or persistent	Tremelimumab	Olaparib
1/2	NCT02571725	PARP-inhibition and CTLA-4 Blockade in BRCA-deficient Ovarian Cancer	BRCA-deficient recurrent platinum sensitive/resistant	Tremelimumab	Olaparib
1/2	NCT02657889	Study of Niraparib in Combination With Pembrolizumab (MK-3475) in Patients With Triple-negative Breast Cancer or Ovarian Cancer (KEYNOTE-162)	recurrent platinum resistant	Pembrolizumab	Niraparib
1/2	NCT02726997	Matched Paired Pharmacodynamics and Feasibility Study of Durvalumab in Combination With Chemotherapy in Frontline Ovarian Cancer	primary	Durvalumab	Carboplatin Paclitaxel
1	NCT02737787	A Study of WT1 Vaccine and Nivolumab For Recurrent Ovarian Cancer	≥2nd remission	Nivolumab	WT1 vaccine
0	NCT02728830	A Study of Pembrolizumab on the Tumoral Immunoprofile of Gynecologic Cancers	primary	Pembrolizumab

#### Nivolumab

Nivolumab is a fully humanized IgG4 monoclonal antibody targeting the PD-1 receptor and is FDA approved for the treatment of melanoma, NSCLC, renal cell carcinoma, and Hodgkin’s lymphoma. A study of nivolumab in recurrent ovarian cancer was the first to be published for this patient population [25]. In this study 20 patients with platinum-resistant ovarian cancer were treated in 2 cohorts either with 1 or 3 mg/kg nivolumab every 2 weeks until progression or up to 48 weeks. Best overall response was the primary endpoint. Grade 3 or 4 adverse events occurred in 8 patients (20%) and two experienced severe adverse events (grade 3 disorientation, gait disorder, fever in 1 patient and grade 3 fever, deep venous thrombosis in the other). The best overall response was 15%. Four patients experienced prolonged disease control (2 patients in each dose cohort) with 2 patients in the 3 mg/kg cohort experiencing a durable complete response (CR). While response rates were similar to what has been seen with chemotherapy in platinum resistant disease, the durable responses are atypical in this disease and a cause for enthusiasm particularly in a very heavily pre-treated population. PD-L1 expression did not significantly correlate with objective response. Fourteen of 16 patients with PD-L1 high expression did not show a response while 1 of 4 patients with low expression was a responder.

#### Pembrolizumab

Pembrolizumab is an anti-PD-1 humanized IgG4 monoclonal antibody FDA-approved for the treatment of melanoma and NSCLC. A non-randomized, multicohort phase Ib study (KEYNOTE-028, NCT02054806) was conducted of single-agent pembrolizumab in ovarian cancer patients [26]. Eligibility requirements included expression of PD-L1 in 1% of tumor nests or PD-L1 expression in stroma. Pembrolizumab 10 mg/kg was given every 2 weeks until progression, intolerable adverse effects or for up to 2 years. Twenty-six patients were treated. Objective response rate was 11.5% with 1 CR, 2 partial responses (PR), and 23% stable disease (SD). Durable responses were noted and the median time to response was 8 weeks.

#### Ipilimumab

Ipilimumab is a recombinant, IgG1 human monoclonal antibody targeting CTLA-4 that is FDA-approved for the treatment of melanoma. In a phase II study of ipilimumab monotherapy in recurrent platinum-sensitive ovarian cancer (NCT01611558), 40 patients were treated with 10 mg/kg ipilimumab every 3 weeks x 4 doses (induction phase) followed by 10 mg/kg every 12 weeks until progression or unacceptable toxicity [27]. Of the 40 who started the study, 38 (95%) did not complete the induction phase because of disease progression (14, 35%), drug toxicity (17, 42.5%), death (1, 2.5%), or other/unreported (6, 15%). Twenty patients (50%) experienced drug-related adverse events of grade 3 or higher. The objective response rate (ORR) was 10.3% [95% confidence interval (CI) 2.9 to 34.2%] by RECIST criteria. Of note, the 10 mg/kg dose is higher than the FDA approved dose for the treatment of unresectable or metastatic melanoma (3 mg/kg) but is equivalent to the dose used for the adjuvant treatment of melanoma.

#### Avelumab

Avelumab is a fully humanized monoclonal anti-PD-L1 IgG1 antibody that does not block PD-1 interaction with PD-L2. In a Phase Ib (NCT01772004, Javelin solid tumor study) [28], 124 patients with refractory or recurrent ovarian cancer (progression within 6 months, or after 2^nd^/3^rd^ line treatment) were treated with 10 mg/kg every 2 weeks until progression or unacceptable toxicity. The median duration of treatment was 12 weeks. Grade 3/4 adverse events occurred in 6.4% of patients and 8.1% of patients discontinued treatment secondary to an adverse event. Twelve patients experienced a partial response for an ORR of 9.7%. Disease control rate (DCR, defined as ORR + SD) was 54%. ORR was 12.3% in PD-L1+ tumors and 5.9% in PD-L1- tumors (based on > =1% threshold). Differences in median PFS and OS were not statistically significant based on PD-L1 expression. There are currently two Phase 3 trials of avelumab for ovarian cancer; one for front-line therapy in combination with carboplatin and paclitaxel (Javelin ovarian 100) and the other for recurrent platinum-resistant disease (Javelin ovarian 200) (Fig. 2).

#### Durvalumab

Durvalumab is an Fc optimized IgG1 monoclonal antibody directed against PD-L1, recently given breakthrough therapy designation by the FDA for PD-L1 positive urothelial bladder cancer. In an ongoing phase I/II study of durvalumab (NCT02484404) in combination with either the PARP inhibitor, olaparib, or the VEGFR inhibitor, cediranib, there was 1 PR in 9 evaluable ovarian cancer patients lasting >6 months with the combination of durvalumab and olaparib and 1 PR in 5 evaluable ovarian cancer patients treated with durvalumab and cediranib [29].

#### Other immune checkpoint inhibitors

Atezolizumab is an Fc-engineered, humanized, non-glycosylated IgG1 kappa monoclonal antibody targeting PD-L1 that is FDA-approved for the treatment of bladder/urothelial carcinomas. Tremelilumab is a fully humanized antibody against CTLA-4. To date no studies have reported outcomes for patients with ovarian cancer treated with atezolizumab or tremelilumab.

Although cross trial comparisons are not feasible given the early stage of development and different trial eligibility parameters, it is remarkable that all of the studies so far have similar ORR (10–15%). This is markedly lower than was seen in early trials of PD-1 inhibitors for Hodgkin’s lymphoma where >65% of patients had a response to treatment and 17–21% achieved a complete response [30, 31], but more consistent with response rates in previously treated patients with melanoma (28%), NSCLC (18%), and renal cell carcinoma (27%) [32]. The phase Ib avelumab study suggests that ovarian cancer patients who have been treated with fewer prior courses of chemotherapy may have a greater benefit from these agents, thus chemotherapy may induce T-cell exhaustion or have other irreversible immunosuppressive effects [33].

Combining the nivolumab and avelumab studies cited above, it is notable that 4 of the 5 patients who experienced durable responses had tumors with clear cell histology [25, 28]. Histology of responding patients on other studies has not been reported. These observations run counter to the prediction that ovarian cancers with clear cell histology would be less likely to respond to PD-1 inhibitors based on low PD-1 expression and low TIL infiltration (Table 2). However, they are particularly intriguing given the characteristic chemorefractory nature of ovarian clear cell carcinomas (OCCC) [34, 35]. Because OCCC are genomically remarkably similar to RCC, it has been postulated that therapies effective for RCC may be similarly effective for OCCC [36]. Nivolumab was recently approved for the treatment of advanced RCC based on the phase III CheckMate 025 trial in which nivolumab showed an ORR of 25% and a 5 month OS benefit over everolimus (ORR 5%) [37]. Whether immune checkpoint inhibitors will result in such dramatic benefits in OCCC remains to be determined in larger cohorts.

### Opportunities to improve outcomes with immune checkpoint inhibitors

#### Identification of predictive biomarkers

A critical need in this field is the development of biomarkers that can predict response to therapy, provide early indication of efficacy, and warn of the development of adverse effects. The most promising have focused on prediction of response to PD-1/L1 therapy. Indications in melanoma trials that tumor PD-L1 expression, density of TILs, and proportion of T cells expressing PD-1 or PD-L1 was associated with response led to the categorization schema outline above in an attempt to identify subsets of melanoma patients who would be most likely to respond to treatment (Table 1) [3, 9]. Further validation is still necessary to determine whether this categorization predicts better outcomes. Individually none of these factors is a reliable predictor of response.

#### PD-L1 expression and TILs

Several studies of anti-PD-1/L1 therapeutic antibodies in multiple tumor types, including melanoma and NSCLC, have suggested that PD-L1 expression is associated with a greater likelihood of benefit [8, 32, 38–40]. These studies typically categorized tumors as PD-L1 positive if at least 5% of tumor cells showed cell-surface PD-L1 staining. While initial studies suggested that PD-L1 negative tumors did not show response [32, 38], subsequent studies in multiple tumor types have shown objective responses in up to 20% of PD-L1 negative tumors [39, 41, 42]. In comparison, the phase 2 nivolumab study in ovarian cancer patients showed that only 2 of 16 patients with high PD-L1 expression showed a response, while 1 of 4 patients with low expression responded [25]. Similarly, the avelumab study showed that even with a staining cut-off level of ≥1% of tumor cells in ovarian cancer, 1 of 17 patients with a PD-L1 negative tumor showed an objective response [28]. Thus, it is unclear whether PD-L1 can be used reliably as a predictive biomarker for anti-PD-1/L1 directed therapy. By contrast, PD-L1 expression status does not appear to influence response to anti-CTLA-4 therapy. In a study of previously untreated melanoma, median PFS (mPFS) in response to ipilimumab was unaffected by PD-L1 status (PD-L1 positive 3.9 months, 95% CI 2.8 to 4.2 months versus PD-L1 negative 2.8 months, 95% CI 2.8 to 3.1 months) while response to nivolumab was influenced by PD-L1 status (14.0 months, 95% CI 9.1 to not reached versus 5.3 months, 95% CI 2.8 to 7.1 in PD-L1 positive versus PD-L1 negative tumors, respectively) [43].

Other attempts at identifying predictive biomarkers have focused on T-cell infiltration. In melanoma, T-cell density, particularly at the invasive tumor border, has been associated with response to anti-PD-1 therapy, however tumors with low T cell density have also shown response [44]. However, a separate study evaluating factors associated with response to anti-PD-1 therapy in multiple solid tumors treated on a phase I clinical trial showed that the presence of TILs did not correlate with clinical outcomes [8]. Response to anti-CTLA-4 therapy has been associated with a more inflamed tumor microenvironment and potential markers include increased expression of the activation marker, inducible T-cell co-stimulator (ICOS), on peripheral blood CD4+ cells and tumor-infiltrating lymphocytes, and an increase in Teff:Treg cell ratio in tumor tissues [45–50]. However no validated predictive biomarkers for CTLA-4 therapy are yet available.

Several factors may account for the difficulty in using receptor expression and T-cell populations as predictive biomarkers. PD-L1 expression and T-cell infiltration are dynamic processes so evaluation of tissue archived at the time of surgery may not reflect the level of expression at the time of recurrence or planned treatment. Small tumor specimens may miss focal expression of PD-L1 or T-cells localized only at the leading edge of the tumor. Scoring of immunohistochemistry for PD-L1 and TILs has not yet been standardized. Additionally, there are challenges associated with the use of different antibodies, fixation and staining techniques, and the subjective interpretation of staining thresholds. Added to this complexity is defining the importance of the subset of cells (immune versus tumor) upon which PD-L1 is expressed. At this point, there is no indication that PD-L1 expression or TIL by IHC should be used as an absolute selection criterion for therapy.

#### Mutational load

Genetic alterations within a tumor (including mutations, DNA rearrangements, deletions, and insertions) have the potential to generate neo-antigens which are associated with clinical response to immune checkpoint therapies. Tumors with higher mutational loads, such as melanoma, NSCLC, and bladder cancer, have shown the greatest response rates to anti-PD-1/PD-L1 therapy, while cancers with relatively low mutation rates (pancreatic and prostate cancers) have shown low response to these therapies [51]. In melanoma, mutational load was associated with the degree of clinical benefit to CTLA-4 blockade and pembrolizumab [52–54]. However, there are patients with low mutational burden who have responded and those with high mutational burden that have not and no specific cut-off point could be determined under which patients would not derive benefit [54].

Mismatched repair (MMR) deficiency, defined by defects in one or more of 6 genes involved in the DNA mismatch repair complex, results in 10–100 fold increases in tumor mutational burden compared to MMR-competent tumors. MSI tumors express high levels of multiple immune checkpoint molecules including PD-1, PD-L1, CTLA-4 and lymphocyte activation gene 3 (LAG3) [55]. Le et al. showed that MSI-high status in colorectal cancer and mismatch-repair deficient non-colorectal cancers was able to predict clinical response to pembrolizumab in a phase 2 trial [56, 57]. There was an ORR of 48% across tumor histologies with 12 month OS and PFS rates of 79 and 54%, respectively. Germline MMR gene inactivation only occurs in ~2% of ovarian cancers, however somatic loss of expression can occur in up to 29% of ovarian cancers [58]. Whether microsatellite instability status (MSI) status may be a predictive biomarker to identify genetic subsets of ovarian tumors with an improved likelihood of response to immune checkpoint inhibition remains to be determined.

In non-MMR deficient ovarian cancers, the predominant genetic abnormality is copy number alteration and mutation rate is generally low [59]. Despite relatively low mutation burdens compared to other cancers, increased neo-antigen presentation may result from other genetic alterations. Patients with BRCA-associated tumors may be more likely to have a higher burden of genetic alterations (copy number alterations, deletions, amplifications) given the role of BRCA in homologous recombination DNA repair [60]. BRCA1-associated ovarian cancers have also been associated with increased intra-tumoral T-cell infiltration [61]. Thus it has been suggested that these patients may derive greater benefit from immune checkpoint inhibitors. Contrary to this hypothesis, no responses were seen in patients with a BRCA mutation in the avelumab phase Ib study (ORR 16% in BRCA-wildtype tumors), DCR was 11.1% in BRCA-mutation carriers versus 48.0% in BRCA-wildtype [28]. Thus at this time, there is no reason to suggest that immunotherapy trials should be limited to this patient population, beyond the stated objective of the study.

#### Functional assays

Other approaches to developing predictive biomarkers include assessing functional capacity of the immune cells within the tumor microenvironment. These approaches include intracellular cytokine staining to measure interferon-gamma signaling and T-cell polyfunctionality, measurement of local inhibitory cytokine production (IL-10, TGF-beta), measurement of T-cell activation or proliferation potential, and T-cell clonality/repertoire [62–69]. However, as of yet, none of these approaches has been validated to be predictive of response to therapy.

#### Combinatorial therapy to improve therapeutic outcomes

Improved therapeutic efficacy has been demonstrated with the combination of anti-PD-1 and anti-CTLA-4 inhibitors. In previously untreated melanoma patients, median PFS was 11.5 months (95% CI 8.9–16.7 months) with nivolumab and ipilimumab compared with 2.9 months (95% CI 2.8–3.4) with ipilimumab and 6.9 months (95% CI 4.3 to 9.5) with nivolumab alone [43]. Interestingly, in patients whose tumors did not express PD-1, median PFS was improved in patients receiving both drugs compared to those who received nivolumab alone; there was no difference in median PFS between these two treatment groups in patients with PD-1 expressing tumors. The frequency of treatment related toxicities was also increased with combination therapy; 55% of patients experienced grade 3/4 events in the nivolumab and ipilimumab group, 16.3% in the nivolumab group, and 27.3% in the ipilimumab group. In mouse models of ovarian cancer, 1/3 to 1/2 of TILs coexpressed PD-1 and CTLA-4 [70]. This subpopulation exhibited poor effector functions with diminished capacity to secrete effector cytokines and proliferate. Dual blockade of PD-1 and CTLA-4 increased T-cell activity and tumor regression. This treatment strategy is currently being evaluated for recurrent ovarian cancer in the NRG Oncology study GY003 (NCT02498600).

Similarly, combining immune checkpoint inhibitors with other anti-neoplastic treatments to enhance therapeutic outcomes is an active area of investigation (see Table 4 for trials for ovarian cancer). Chemotherapy, radiotherapy, tyrosine kinase inhibitors, and epigenetic modulators may be synergistic adjuncts to immunotherapy through their ability to increase tumor immunogenicity [71–74]. A particular area of interest for ovarian cancer is the combination of immune checkpoint inhibitors with anti-angiogenic agents and/or PARP inhibitors. Both anti-angiogenic agents and PARP inhibitors influence the ovarian cancer immune microenvironment in in vivo models and combination therapy is supported by preclinical studies [75–78]. Clinical trials of combination therapy are ongoing, so far phase I results of durvalumab/cediranib and durvalumab/olaparib (NCT02484404) show the combinations are feasible. It will be necessary to develop individualized approaches to determine which treatment strategy is most likely to be effective for individual patients.

### Management considerations with immune checkpoint inhibitors

#### Immune-related toxicities

Related to their mechanism of action of impairing T-cell inhibition, immune checkpoint inhibitors can cause a loss of self-tolerance and thus the development of immune-related adverse effects (irAEs). While the frequency of irAEs with these agents is common (~60% with anti-CTLA-4 therapy and 40% with anti-PD-1/L1 therapy), in general serious toxicity [grade 3–5 using the Common Terminology Criteria for Adverse Events (CTCAE)] is more likely with anti-CTLA-4 therapy (>40%) than with anti-PD-1/L1 therapy (~5%) [32, 79, 80]. While the immune side effects could involve any organ system, the most common irAEs with both anti-CTLA-4 and anti-PD-1/L1 therapy are dermatologic (rash, pruritis), gastrointestinal (diarrhea), rheumatologic (arthralgia, arthritis, myalgia, myositis), endocrine disorders (thyroiditis, hypothyroidism, hypophysitis), and infusion-related reactions. Serious gastrointestinal toxicities, such as immune-mediated colitis and hepatitis, are more likely with anti-CTLA-4 therapy. Involvement of the following organ systems is much less common, especially with anti-PD-1/L1 pathways inhibitors, but have been observed including: pulmonary (pneumonitis, sarcoidosis), hematologic (hemolytic anemia, aplastic anemia, neutropenia), ocular (uveitis, conjunctivitis), cardiac (myocarditis, pericarditis), neurologic (myasthenia gravis, Guillan Barre, Bell’s palsy, posterior reversible leukoencephalopathy, and aseptic meningitis). While irAEs associated with these therapies are generally reversible, without prompt management they can evolve into life-threatening conditions. Early recognition of the development of these events is critical to prevention of the progression to severe adverse effects. Vigilance, both on the part of the patient and the treating physician, is necessary to identify subtle developing signs of irAEs and specialty consultation may be necessary when irAEs are suspected but not definitive. Immune-related AEs can develop at any time during treatment and even after discontinuation of therapy. However, the onset of irAEs follows a characteristic pattern of development. For the CTLA-4 inhibitor, ipilimumab, most irAEs develop during the initial induction period (usually 4 doses given every 3 weeks). Dermatologic reactions more commonly occur early during treatment, frequently during the first few weeks, while diarrhea and colitis develop later. Endocrine disorders may be late effects, frequently developing between 7 and 20 weeks after initiation of treatment and sometimes being identified after discontinuation of treatment [81]. Combining CTLA-4 inhibitor and PD-1 inhibitors, while significantly increasing response also results in substantially more irAEs.

Spain et al. recently provided a substantive review of the management of irAEs [82]. Briefly, management is dependent on the severity of the event, usually graded using CTCAE. All references to grade in this article will be using CTCAE version 4.0 [83]. Typically grade 1 symptoms can be monitored and may not require interruption of therapy. For Grade 2 symptoms, the immune checkpoint inhibitor therapy should be withheld until symptoms improve and treatment with an immunomodulatory medication may be considered. Immune-related AEs with a higher risk of resulting in serious organ dysfunction (such as colitis, hepatitis, pneumonitis, nephritis, neurologic symptoms) likely warrant initiation of corticosteroid treatment early (Grade 2) rather than waiting for symptoms to worsen. When the severity of the irAE warrants the reversal of inflammation (≥ Grade 3), corticosteroids are the first immunomodulatory medication to be administered. Careful and timely monitoring of response to steroid therapy is required to identify steroid-refractory cases. In these situations, the use of more potent immunomodulatory agents may be necessary such as the anti-TNF-alpha antibody infliximab, the anti-metabolite mycophenylate mofetil, anti-thymocyte globulin, and/or calcineurin inhibitors (i.e. tacrolimus and cyclosporine). Consultation with, and if indicated hospitalization at, a center familiar with steroid-refractory immune checkpoint inhibitor irAEs is advised.

#### Steroid use during treatment

Because of the effect of steroids on inhibiting T-cell activation, patients receiving supraphysiologic doses of corticosteroids have generally been excluded from trials of immune checkpoint inhibitors. In the most comprehensive retrospective review on the subject, Horvat et al. evaluated the effect of initiation of immunomodulatory agents for ipilimumab irAEs in melanoma patients [84]. Of the 298 patients, 85% experienced an irAE of any grade, 35% required corticosteroid treatment, and 10% required anti-TNFalpha therapy. Overall survival and time to treatment failure (median 5.7 months) was not affected by the occurrence of irAEs or the use of systemic corticosteroids. Continued anti-tumor activity has been observed in patients treated with high-dose steroids for irAEs [85]. However, data on the effects of steroid use on immune checkpoint inhibitor efficacy are still limited. Thus, it is recommended to avoid prophylactic steroids and limit therapeutic steroids as needed.

#### Measurement of response: specific immune related criteria

One of the challenges faced in assessing the therapeutic value of these agents is determining the most appropriate measurement of efficacy. While response rates to immune checkpoint inhibitors as single agents is relatively low, the impressive duration in responding patients suggests that overall survival may be a better measure of efficacy. In fact, there have been patients who achieve long-term survival benefit without evidence of clinical response [53]. In addition, early studies noted that some patients had responses after initial apparent progression of disease, while others showed a mixed response or new lesions despite an overall decrease in tumor burden. Since these response patterns were not adequately captured by RECIST1.1, new immune-related response criteria (irRC) were developed to specifically accommodate the response patterns seen after treatment with immune checkpoint inhibitors [86]. Unlike in RECIST1.1, new lesions do not automatically signal progression and apparent progressive disease must be confirmed 4 weeks after initial assessment to qualify for true progression. In melanoma patients treated with either ipilimumab or pembrolizumab, ‘pseudoprogression’ occurred in ~ 7% of patients and has been attributed to peritumoral lymphocyte infiltration or delayed immune activity [87–89]. RECIST1.1 was noted to underestimate the benefit of pembrolizumab in this population by up to 15% [88]. However, because this phenomenon occurs relatively infrequently, many studies continue to use RECIST1.1. In order to adequately assess efficacy across studies, it will be necessary to harmonize response assessments across studies and identify more refined radiographic or biologic markers of early efficacy. In addition, the development of other immune-specific clinical trial endpoints may be necessary to account for prolonged duration of response after initial progression [90].

Rates of pseudoprogression in ovarian cancer have not been reported, but trials to date suggest it occurs less frequently than in melanoma [25, 26, 28]. Thus, in contrast to the management of melanoma, progression by RECIST1.1 is likely true tumor progression. While treatment beyond progression is sometimes considered in melanoma patients until true progression is confirmed, further treatment after progression in ovarian cancer patients may carry additional risks as peritoneal implants could progress to cause bowel obstruction.

## Conclusions

The advent of immune checkpoint inhibitors has stimulated increased enthusiasm for immune-oncology. In ovarian cancer, while there is compelling data that the immune microenvironment influences outcomes, early results of clinical trials of immune checkpoint inhibitors suggest limited tumor response. Strategies to improve treatment outcomes and minimize immune-related toxicities are necessary and will likely require individualized approaches. There are multiple areas in which the cancer-immune system interaction can fail to result in adequate anti-tumor activity. To better understand these areas, the development of biomarkers to determine those therapies active in an individual tumors, so called ‘personalized immunotherapy’, are critical. Some have suggested the use of the “cancer immunogram” to describe individual tumor:immune system interactions [91]. Biomarker guided clinical trials will be necessary to tailor these approaches to ovarian cancer patients. We anticipate that tumor genomic profiling will need to be integrated with immune profiling to provide a more comprehensive understanding of an individual patient’s tumor leading to improved treatment selection and sequencing.

## Abbreviations

APC: Antigen presenting cell
CI: Confidence interval
CTCAE: Common Terminology Criteria for Adverse Events
CTLA-4: Cytotoxic T lymphocyte-associated protein 4
DC: Dendritic cell
Ig: Immunoglobulin
IL-10: Interleukin 10
irAE: Immune-related adverse event
irRC: Immune-related response criteria
LAG3: Lymphocyte activation gene 3
MDSC: Myeloid derived suppressor cell
MHC: Major histocompatibility complex
MMR: Mismatched repair
mPFS: Median PFS
MSI: Microsatellite instability
NSCLC: Non-small cell lung cancer
OCCC: Ovarian clear cell carcinoma
ORR: Objective response rate
OS: Overall survival
PD-1: Programmed cell death protein 1
PD-L1: PD-1 ligand 1
PD-L2: PD-1 ligand 2
PFS: Progression free survival
RCC: Renal cell carcinoma
RECIST: Response evaluation criteria in solid tumors
T_eff_: Effector T cells
TGFbeta: Transforming growth factor beta
TIL: Tumor-infiltrating lymphocytes
TNFalpha: Tumor necrosis factor alpha
T_regs_: Regulatory T cells
